# RRMdb—an evolutionary-oriented database of RNA recognition motif sequences

**DOI:** 10.1093/database/bay148

**Published:** 2019-01-16

**Authors:** Martyna Nowacka, Pietro Boccaletto, Elzbieta Jankowska, Tomasz Jarzynka, Janusz M Bujnicki, Stanislaw Dunin-Horkawicz

**Affiliations:** 1 Laboratory of Bioinformatics and Protein Engineering, International Institute of Molecular and Cell Biology in Warsaw, Warsaw, Poland; 2 Laboratory of Structural Bioinformatics, Centre of New Technologies, University of Warsaw, Warsaw, Poland

## Abstract

RNA-recognition motif (RRM) is an RNA-interacting protein domain that plays an important role in the processes of RNA metabolism such as the splicing, editing, export, degradation, and regulation of translation. Here, we present the RNA-recognition motif database (RRMdb), which affords rapid identification and annotation of RRM domains in a given protein sequence. The RRMdb database is compiled from ~57 000 collected representative RRM domain sequences, classified into 415 families. Whenever possible, the families are associated with the available literature and structural data. Moreover, the RRM families are organized into a network of sequence similarities that allows for the assessment of the evolutionary relationships between them.

## Introduction

The RNA-recognition motif (RRM) domains are important players in the regulation of development (1), signaling (2), gene expression (3) and cell differentiation (4). A typical RRM domain consists of approximately 90 amino acid residues that fold into a four-stranded β-sheet with two α-helices packed against it (β1–α1–β2–β3–α2–β4). In most cases, the RNA recognition by RRMs is sequence-specific and occurs via two conserved motifs RNP1 and RNP2 localized in β3 and β1, respectively (5). However, a number of exceptions were identified where bona fide RRM domains bind RNA molecules in a different manner (6) or recognize other molecules (7) such as proteins (8) or DNA (9). A single canonical RRM domain recognizes an RNA fragment comprising up to eight ribonucleotides; however, many RNA-binding proteins contain more than a single RRM domain, thus extending the number of recognized ribonucleotides (10). Moreover, the canonical RRM fold can be `decorated’ with additional structural elements such as β-hairpins (11), β-strands (12) or α-helices (13) that contribute to the RNA recognition.

The evolution of RRM domains has been investigated in the context of various protein families. For example, structural studies of the heterogeneous nuclear ribonucleoprotein L (hnRNP L), a protein containing four RRM domains, showed that all four RRMs of hnRNP L contain a functional C-terminal extension (the so-called ICC motif), while both the second and third RRM domains additionally possess fifth β strand (12). Bioinformatics analyses of hnRNP L homologs from various organisms revealed that the acquisition of the unstructured ICC motif was a prerequisite for the emergence of the fifth β strand. Conceptually similar works have focused on the evolution of other RRM-containing protein families such as La-motif superfamily (14), serine/arginine-rich splicing factors (SRSF) (15, 16) and other splicing factors (16). However, to the best of our knowledge, no comprehensive analysis of all RRM domain sequences and their mutual similarities has been performed. Bearing this in mind, we have developed RRMdb, a publicly available database that classifies all known RRM domains into 415 families. The families are associated with the relevant literature, sequence and structural data and organized into a network according to their pairwise sequence similarity. Using the database, the user can quickly assign RRM domains in a given sequence and obtain detailed descriptions of the identified and evolutionarily related RRMs. We provide the RRMdb database as a tool both for the experimentalists searching for basic knowledge about RNA-binding proteins with RRM domains as well as for those studying the evolution of RRM domains.

## Database implementation

Based on the SCOP (17) and PFAM (18) databases a representative set of RRM core (β1–α1–β2–β3–α2–β4) domain sequences was constructed. These sequences were used to search the NCBI non-redundant protein database using PSI-BLAST (19) (three iterations) and all the obtained sequences were collected and filtered to 90% sequence identity with CD-HIT (20), resulting in a database of 57 471 sequences. These sequences were clustered into 415 families based on all-vs.-all BLAST comparisons using the Markov Cluster (MCL algorithm) (21). Specifically, BLAST e-values were transformed using −log_10_ function and values greater than 30 were capped to 30. MCL was started with the inflation parameter set to 2.0. To verify the robustness of the clustering procedure we performed two tests. First, we generated 10 000 artificial natural-like protein sequences using NullSeq (22) (amino acid usage frequencies were defined based on known RRM domains). Clustering of these artificial sequences together with natural RRM sequences revealed that none of the artificial sequences grouped with RRM sequences, indicating that the clustering cut-offs ensure robust discrimination between RRM and non-RRM sequences. Second, we calculated the Silhouette Coefficient (SC) (23) for each clustered RRM sequence. The best value of SC is 1, and the worst value is −1. Values near 0 indicate overlapping clusters, whereas negative values generally indicate that a sample may have been assigned to the wrong cluster. We found that only 9% of sequences have SC below 0, whereas 72% have SC 0.5 or greater. Considering the continuous character of protein sequence space, it is to be expected that not all sequences can be unambiguously assigned to a single cluster and that some could lie between two or more clusters.

**Figure 1:**
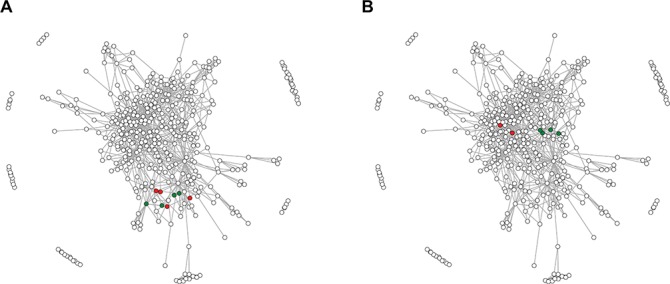
The network of RRM domain families. Points correspond to 415 RRM families defined in this study, whereas edges connect significantly (e-value of profile–profile comparisons below 1e-5) similar families. (**A**) Green points indicate four families encompassing RRM domains of the hnRNP L; red points indicate RRM families from related hnRNP L-like proteins. (**B**) Green and red points indicate RRM families of SRSF proteins—for details see the text.

For each family, a multiple sequence alignment was generated using MUSCLE (24), corrected manually and used to calculate a Hidden Markov Model (HMM) using hhmake (25). The HMMs were aligned in all-vs.-all fashion using hhsearch (`-ssm 0’ flag was used to disable secondary structure scoring during alignment) (25). All hhpred output files were handled with the aid of CSB package (26). The resulting e-values were used to generate a network in which nodes represent RRM families and edges denote significant (e-value < 1e-5) similarities between them (Figure 1). A comprehensive survey of literature resulted in a list of 503 publications from which data about the specificity, function and structure of 297 RRM-containing proteins were extracted. The 297 protein sequences were scanned with BLAST and the matching RRMdb families were associated with the corresponding literature data. Sequence fingerprints of the RNP1 and RNP2 motifs were calculated based on 50 RRM domains interacting in a canonical and non-canonical manner with the RNA substrate (27). These fingerprints are used to detect potential RNP1 and RNP2 motifs in a user-provided sequence.

## Database web interface

A web interface for the RRMdb was implemented in Django and is available at http://iimcb.genesilico.pl/rrm/. The database can be searched using a protein sequence to identify and label the RRM domains according to the definition of 415 families. For each identified RRM domain family, RRMdb provides information about the N- and C-terminal residue within the full-length protein sequence, conserved residues, positions of the potential RNP1 and RNP2 motifs and an alignment between the user-provided sequence and a matching RRM family profile. Moreover, the search results page includes links to the relevant publications, structures, model proteins and mappings to RBPDB (28), ECOD (29) and PFAM (18) databases.

Owing to the network-based representation of the RRM families, the RRMdb database not only returns information about the individual RRM domains identified in a given protein but also provides details about evolutionarily related families and families that are found together with the one of interest within multi-RRM proteins. Alternatively, the database can be accessed by browsing the network (`explore’ tab) or the list of all families (`browse’ tab).

## Application of the database—case studies

Multi-RRM proteins are common regulators of alternative splicing [e.g., polypyrimidine tract-binding protein (PTB), Sxl] and the study of their origin and mutual relationships is important to understand how proteins increase their RNA-binding specificity and affinity. There are three possible evolutionary scenarios that could have led to the emergence of a multi-RRM protein (and to an orthologous family of multi-domain RRMs): (i) duplication of RRM domain(s) within a protein family, (ii) recombination or gene fusion resulting in domain shuffling between RRM-containing proteins and (iii) a combination of these two aforementioned events. The duplication might be supposed if RRM domains in a given protein family are more similar to each other than to any other RRM domains, whereas the recombination should be taken into account if RRM domains in a given protein family are more similar to other families than to each other. To distinguish between these two possibilities, it is necessary to assess the similarity between RRM domain families. Relying only on a pairwise sequence similarity value can be misleading; for example, two families that display low sequence similarity (i.e., lower than the average similarities between all the other families) may be, at the same time, their closest relatives that descended from a common ancestor. This problem can be addressed by applying an approach in which the evolutionary distance is approximated as the shortest path connecting two given RRM families in the network (Figure 1). The shortest path is defined as a number of intermediate families that have to be traversed to connect the two families, and it assumes the value of zero if the two families are directly associated. Such an approach ensures that RRM families that display a low sequence similarity but share proximity on the graph will be considered as closely related. To highlight the functionality of the RRMdb, we analyzed two families of multi-RRM proteins, namely, the aforementioned hnRNP L-like proteins and SRSF.

The hnRNP L is a protein involved in many aspects of RNA metabolism and contains four RRM domains. The hnRNP L protein is a founding member of a family encompassing proteins with similar domain composition such as PTB, neural PTB and PTB 3 (Rod1). Despite the fact that the homology between the four RRMs of hnRNP L is barely detectable with BLAST, a recent study (12) has suggested that they have all evolved from a single common ancestor that already contained the ICC. In the graph representation, the four hnRNP L RRMs (families 46, 33, 68 and 223; green dots in Figure 1A) and other ICC-containing RRMs of hnRNP L-like proteins (families 375, 393, 353 and 103; red dots) are grouped together (average shortest path ~1), indicating that fast and automated annotation by the RRMdb database provides conclusions that are in accordance with the results of otherwise laboriously manual investigations.

Typical SRSFs contain one or two RRM domains followed by a single low-complexity SR domain. In the work by Califice and colleagues (15) SRSF proteins were classified into four groups (A, B, C and D) based on the phylogeny of the RRM domain. Moreover, the authors have suggested that these RRM domains originate from a single common ancestor. Indeed, the families 70 and 18 encompassing RRM domains of single-RRM SRSFs from group B defined in (15) and families 73 and 126 containing RRM1 domains of double-RRM SRSFs (group C) are clustered together in the network (the average shortest path ~0; Figure 1B; green dots). However, we found that RRM domains of single-RRM SRSFs from groups A (family 28) and D (family 57) are localized in different regions of the network (the average shortest path between groups B/C and A/D is ~2), suggesting an alternative evolutionary scenario in which the SRSF proteins have acquired their RRM domains independently. This example also shows that results provided by the RRMdb are consistent with the results of classical phylogeny but at the same time provide additional clues on the RRM domain evolution.

RRMdb is proposed as a computational resource with which to study these and related events and to elucidate the complex evolution of RRM domains and RRM domain-containing proteins.

## Notes

**Database URL**: http://iimcb.genesilico.pl/rrm
